# Heterogeneous HIV Testing Preferences in an Urban Setting in Tanzania: Results from a Discrete Choice Experiment

**DOI:** 10.1371/journal.pone.0092100

**Published:** 2014-03-18

**Authors:** Jan Ostermann, Bernard Njau, Derek S. Brown, Axel Mühlbacher, Nathan Thielman

**Affiliations:** 1 Duke Global Health Institute, Duke University, Durham, North Carolina, United States of America; 2 Center for Health Policy and Inequalities Research, Duke University, Durham, North Carolina, United States of America; 3 Community Health Department, Kilimanjaro Christian Medical College, Moshi, Tanzania; 4 Brown School, Washington University in St. Louis, St. Louis, Missouri, United States of America; 5 Stiftungsinstitut Gesundheitsökonomie und Medizinmanagement, Hochschule Neubrandenburg, Neubrandenburg, Germany; 6 School of Medicine, Duke University, Durham, North Carolina, United States of America; Rollins School of Public Health, Emory University, United States of America

## Abstract

**Background:**

Efforts to reduce Human Immunodeficiency Virus (HIV) transmission through treatment rely on HIV testing programs that are acceptable to broad populations. Yet, testing preferences among diverse at-risk populations in Sub-Saharan Africa are poorly understood. We fielded a population-based discrete choice experiment (DCE) to evaluate factors that influence HIV-testing preferences in a low-resource setting.

**Methods:**

Using formative work, a pilot study, and pretesting, we developed a DCE survey with five attributes: distance to testing, confidentiality, testing days (weekday vs. weekend), method for obtaining the sample for testing (blood from finger or arm, oral swab), and availability of HIV medications at the testing site. Cluster-randomization and Expanded Programme on Immunization (EPI) sampling methodology were used to enroll 486 community members, ages 18–49, in an urban setting in Northern Tanzania. Interviewer-assisted DCEs, presented to participants on iPads, were administered between September 2012 and February 2013.

**Results:**

Nearly three of five males (58%) and 85% of females had previously tested for HIV; 20% of males and 37% of females had tested within the past year. In gender-specific mixed logit analyses, distance to testing was the most important attribute to respondents, followed by confidentiality and the method for obtaining the sample for the HIV test. Both unconditional assessments of preferences for each attribute and mixed logit analyses of DCE choice patterns suggest significant preference heterogeneity among participants. Preferences differed between males and females, between those who had previously tested for HIV and those who had never tested, and between those who tested in the past year and those who tested more than a year ago.

**Conclusion:**

The findings suggest potentially significant benefits from tailoring HIV testing interventions to match the preferences of specific populations, including males and females and those who have never tested for HIV.

## Introduction

HIV testing is the critical first step for accessing HIV care and prevention interventions, including antiretroviral therapy. Universal testing and immediate treatment of HIV-infected individuals could help to reduce the HIV epidemic substantially [1]–[4]. Yet, HIV testing rates in sub-Saharan Africa remain low and do not approximate the thresholds required by test-and-treat models to affect HIV incidence [1], [5]–[8]. For example, in Tanzania, only 55% of women and 40% of men have ever tested for HIV, and less than one-third tested within the previous 12 months [9]. The determinants of limited testing uptake are poorly understood [10]–[14]. While vaguely-defined and difficult-to-address barriers such as fear and stigma contribute to low testing uptake, it is possible that the characteristics of current testing options may not align well with population preferences for testing.

To date, assessments of HIV testing preferences in sub-Saharan Africa have typically focused on the acceptability of specific venue-based testing options, such as home-based testing [11], [15], [16], provider-initiated testing [17]–[20], workplace testing [21], [22], or mobile testing [23], [24], each without consideration or offer of other options. Results from such narrow assessments do not probe the potential diversity in testing preferences of target populations; nor do they characterize testing options that might maximize uptake among heterogeneous risk groups. Enhanced understanding of the variations in HIV testing preferences may form the basis for better-informed, evidence-based HIV testing policies and practice.

One survey tool that is well suited for characterizing population preferences for HIV testing is the discrete choice experiment (DCE). Grounded in the economic theory of utility maximization and assuming a behavioral framework with testable predictions, DCEs allow researchers to describe how individuals value selected features of services or goods by asking them to state their choices over different hypothetical alternatives [25]. This approach has been used increasingly to elicit individuals' stated preferences for diverse health services and goods, such as colorectal cancer screening [26], vaccines [27], [28], contraceptives [29], and cancer treatment [30], among others [31]–[33].

HIV testing options can be deconstructed by their various attributes, such as counselor characteristics (e.g. age, gender, experience), test administration (e.g. finger stick, venipuncture, oral swab), counseling time, or travel time. Preferences for specific testing options may also be associated with concerns regarding stigma and confidentiality, the accuracy of test results, or past testing experiences [24], [34]–[37]. How individuals value these characteristics as they evaluate testing options and which characteristics most influence an individual's decision to test are unknown. Phillips, et al. applied DCE methods to measure preferences for HIV testing in San Francisco among clients presenting to public testing sites [38], but to our knowledge no DCE has investigated HIV testing preferences in sub-Saharan Africa.

In this study, we use DCE methodology to describe HIV testing preferences of a population-based sample of residents of the Kilimanjaro Region of Tanzania.

## Methods

### Ethics statement

Study activities were approved by the Institutional Review Boards of Duke University Health System and Kilimanjaro Christian Medical University College, and by the National Institute for Medical Research in Tanzania. Written informed consent was obtained from each participant.

### Setting

The *HIV Testing Preferences in Tanzania* (TP-TZ) study was conducted in Moshi, the administrative capital of the Kilimanjaro Region in Northern Tanzania. Moshi is an appropriate setting in which to assess HIV testing preferences for several reasons: (a) there are numerous HIV testing sites within a well-circumscribed area; (b) with 36% of women and 48% of men never having tested [39], there remains an ongoing need to increase testing in the region; and (c) the context of HIV testing in this region is well understood by our research team [24], [40]–[49].

### Development of the discrete choice experiment

**Figure 1:**
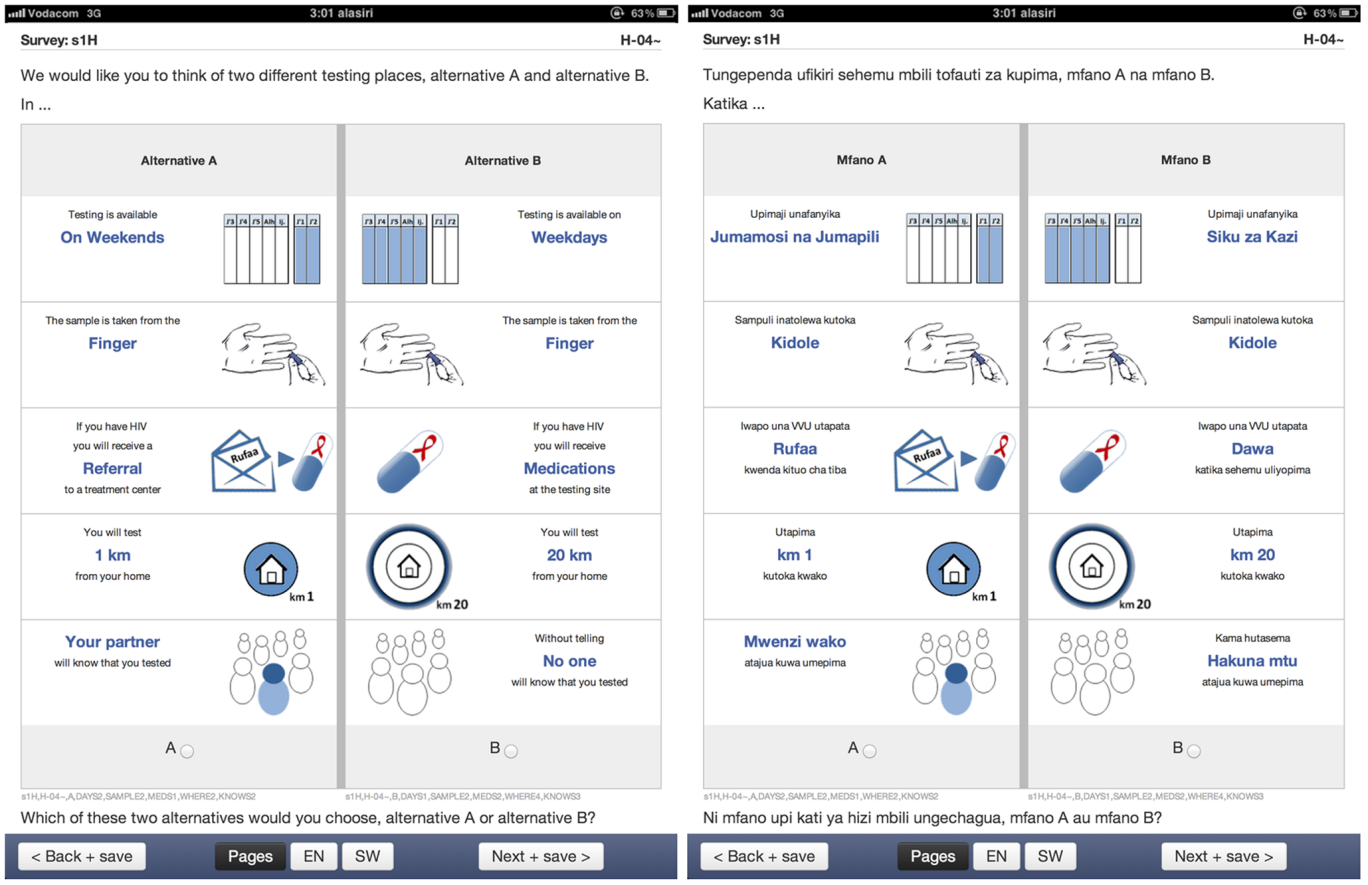
Sample choice task. Choice task shown to participants during the iPad-based presentation of the discrete choice experiment, in English (left) and Kiswahili (right).

DCE development began with a review of prior literature on HIV testing, especially from the Moshi area [24], [41]–[45], [47]–[49], followed by in-depth interviews (IDIs), focus group discussions (FGDs), and pre- and pilot-testing of questionnaires. IDIs with a convenience sample of 8 community members were used for broad conceptualization and the development of an attribute list. Four FGDs with a total of 33 participants (range 7 to 9 persons per group, invited through house-to-house contact in the study area, and stratified by gender and prior HIV testing status) were used to expand and prioritize the attribute list, conduct ranking exercises, and identify plausible values for all attributes, referred to as attribute levels. Graphics were developed to facilitate the pictorial presentation of choice tasks. Two draft paper surveys, refined through iterative pre-tests with 19 community members, and pilot-tested with convenience samples of 21 community members each, were used to further narrow the attributes included in the final version of the survey. The final survey was programmed into an electronic data collection format for presentation on iPads, and pretested with an additional 8 community members. The attributes and levels are shown in **Table 1**; a sample choice task is shown in **Figure 1**.

**Table 1:** Attributes, attribute levels, and results of the direct assessment of preferences (N = 486).

Attribute	Label	Description 1	Most preferred 2	Least preferred 2
			(%)	(%)
Distance	Home	You will test at your home	53.3	22.4
	1 km	You will test in your neighborhood, 1 km from your home	30.9	7.0
	5 km	You will test elsewhere in town, 5 km from your home	12.8	1.9
	20 km	You will test out of town, 20 km from your home	3.1	68.7
Confidentiality	Many people	Without telling, many people who know you will know that you tested	19.1	59.7
	Spouse	Your partner will know that you tested, even without telling	45.9	8.2
	No-one	Without telling, no one whom you know will know that you tested	35.0	32.1
Testing days	Weekdays	Testing is available on weekdays, Monday through Friday	52.3	47.7
	Weekends	Testing is available on weekends, Saturday and Sunday	47.7	52.3
Type of sample	Arm	The sample is taken from the arm	45.3	24.3
	Finger	The sample is taken from the finger	41.8	6.2
	Mouth	The sample is taken from the mouth	13.0	69.5
Services if HIV positive	Referral	If you have HIV you will receive a referral to a treatment center for medications	29.8	70.2
	Medications	If you have HIV you will receive medications at the testing site	70.2	29.8

1 Shortened descriptions were used after the completion of training tasks and the comprehension test (see e.g., Figure 1).

2 Most and least preferred levels of each attribute, without requiring any trade-offs.

### Experimental design

The attributes and levels of the DCE define a large number of possible combinations: 144 possible tests (4*3*2*3*2 attribute-level combinations) which can be combined as 10,296 unique pairs (144*143/2). However, under standard assumptions [50], one can estimate preferences over the range of attributes from a much smaller number of tasks using a fractional factorial design. A *D*-efficient statistical design of 72 choice tasks, allocated as 8 blocks of 9 tasks each, was identified using NGene software [51]. As per current best practice for DCE data the design was optimized for a mixed logit model [52], with additive, categorical terms for each attribute level; parameter estimates from conditional logit analyses of the pilot data were used as priors. Participants were randomly assigned to a block, with the order of choice tasks and testing options within choice tasks (right/left position) randomized within participants. To improve statistical efficiency, the experimental design was updated after enrollment of approximately half of the participants (N = 232 of 486). Estimates from a mixed logit model of the stated choices from the first 202 participants were used as priors in the identification of a new *D*-efficient design. Such an update process has been recommended by others [53], [54] and can improve the precision of the estimates. Interim results suggesting a linear effect of distance and an interaction between distance and availability of medications at the testing site were incorporated into the updated design. Block randomization of the attribute order was also added, with participants randomized into the attribute order shown in **Figure 1**, or an alternate order in which distance and confidentiality were introduced last.

### Survey administration

Surveys were administered between September 2012 and February 2013 by trained research assistants. Interviews were conducted in participants' homes in their native language, Kiswahili. All items were read aloud to participants to ensure that persons of varying degrees of literacy comprehended the material. Literacy [9] and visual acuity [55] were assessed to ensure that participants could follow the presentations of choice tasks. Both graphics and differentiated fonts where used in the visual presentation of the tasks on iPads (**Figure 1**). Participants were provided an incentive of 3,000 Tanzanian Shillings (approximately 1.80 U.S. Dollars).

The DCE was introduced through several orientation screens in which the interviewer provided light assistance to the respondent, as needed, in understanding the tasks. Each attribute and level was individually described and visually depicted, and respondents were asked to state their most and least preferred levels for each attribute. Results of these direct (or unconditional) assessments of preferences were used to dynamically generate two learning tasks that required respondents to simultaneously evaluate multiple attributes in a forced trade-off. Similarly, a comprehension test asked participants to choose between an alternative that combined all their most preferred attribute levels and an alternative that combined all their least preferred attribute levels. Finally, respondents completed the DCE choice tasks. A supplemental paper survey assessed participants' sociodemographic and other characteristics and history of HIV testing (**Appendix S1**).

### Sampling

**Figure 2:**
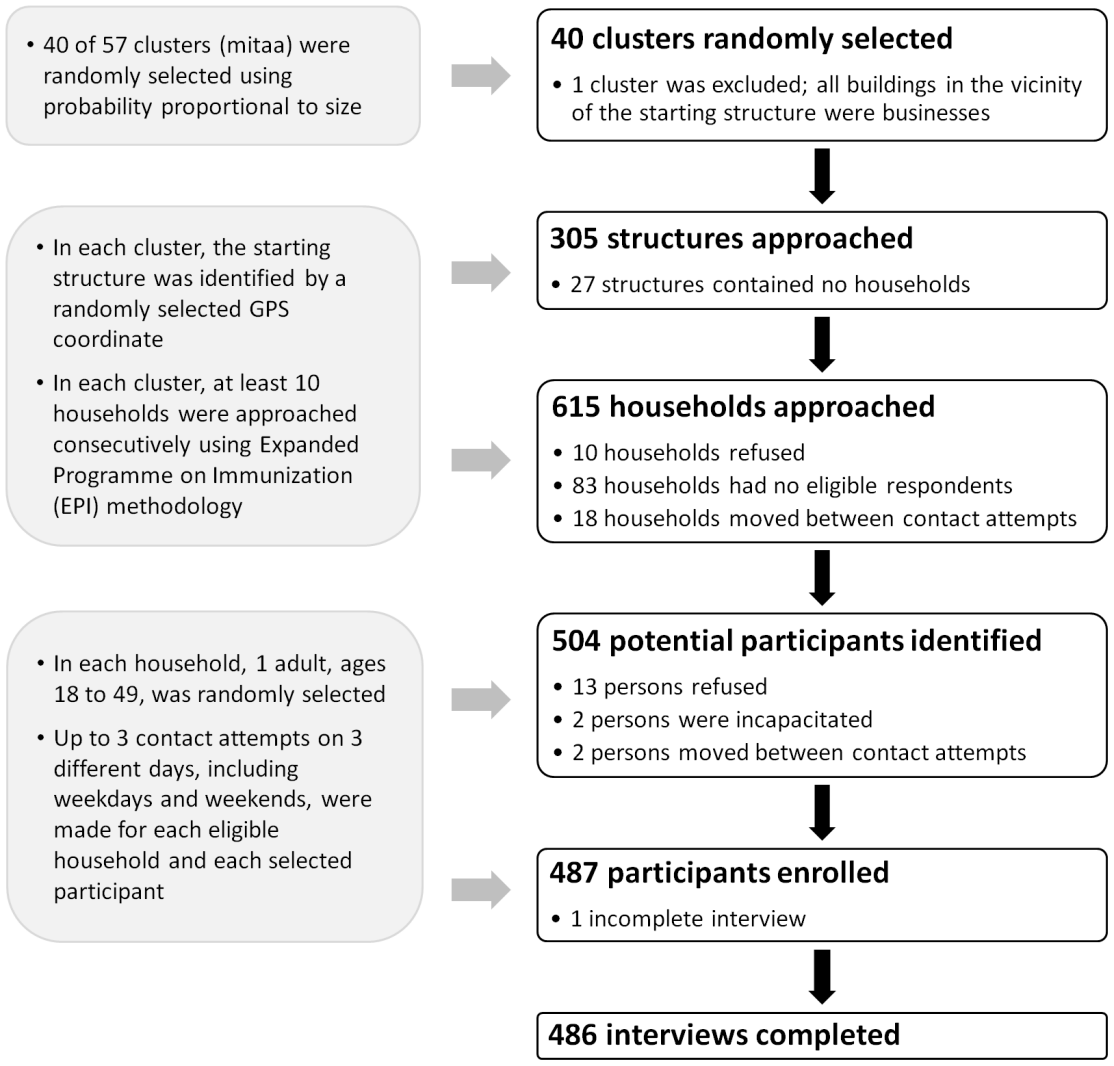
Participant enrollment. Flowchart summarizing enrollment of a random community sample for participation in the discrete choice experiment.

Sampling and enrollment are summarized in **Figure 2**. Forty *mitaa* (singular: *mtaa*, an administrative area translated as ‘neighborhood’) within the district boundaries of Moshi Municipality were randomly selected using probability proportional to size. Within the boundaries of each *mtaa*, 5 geographic coordinates were randomly generated and imported into Google Earth [56]. The first coordinate to fall on a structure was selected as the starting point for participant enrollment. If no point fell on a structure, the structure closest to any of the 5 coordinates was chosen as the starting point. iPad devices and satellite image maps [57] were used to locate the starting point. From each starting point, neighboring structures were iteratively approached using *Expanded Programme on Immunization (EPI)* methodology [58] until 10 participants were enrolled or the boundary of the *mtaa* was reached. Structures identified as clearly non-residential were excluded. One cluster contained only non-residential buildings in the vicinity of the starting structure and was excluded; in one cluster only 5 residential structures were identified. In multi-household structures, all households were approached. In each household, one adult, ages 18–49, was randomly selected using a randomization table with the last digit of the calendar day as the seed. Separate contact attempts on up to 3 different days, including weekdays and weekends, were made for each household and each randomly selected participant. Multiple return visits, aimed at minimizing selection biases due to non-availability of households or participants, increased the average enrollment per cluster to 12.5 participants (range 3 to 23).

### Quality assurance

Strict protocols were developed for all aspects of research implementation, and various elements of data collection were assessed in real or near-real time to ensure high quality of the data. DCE data were uploaded at least daily using the iPad's mobile internet connection and shared with other members of the international study team. Shadowing of each interviewer by another interviewer occurred on a biweekly basis; selected DCE interviews were audio-recorded and reviewed by other members of the study team. The time to complete each choice task (median 44 seconds; inter-quartile range 38–53 seconds) was recorded by the iPad. A principal investigator regularly accompanied field interviewers to the field or communicated with them by phone to ensure adherence to study protocols.

### Statistical analysis

Student's *t*, chi-squared, and Fisher's exact tests were used to assess the statistical significance of gender differences in participants' demographic and other characteristics and HIV testing history. Patterns of choices were analyzed in Stata 12.1 [59] using gender-specific mixed effects logit models [60] with categorical effects coded explanatory variables [61] and correlated random coefficients, which were assumed to be normally distributed. The significance of differences between the levels of an attribute and their joint significance were assessed using Wald tests.

Additional gender-specific mixed logit models included interactions between attribute levels and binary variables describing participants' HIV testing history (previously tested for HIV vs. never tested), and, among those who had tested, the time of their last test (in the past year vs. more than 1 year ago), respectively. Wald tests assessed the statistical significance of the interaction terms. To facilitate convergence of these models, interaction parameters were treated as fixed rather than random coefficients.

## Results

### Sample characteristics

Characteristics of study participants are shown in **Table 2**. Two-thirds of the participants (67%) were female, nearly half (47%) were married, and 46% had more than a primary school education. There were significant gender differences in marital status (p<0.001) and educational attainment (p<0.001). For both men and women, approximately half of the participants were either employed or self-employed. Nearly 40% of males were students and 29% of women were housewives. The number of lifetime sexual partners was higher among men than women (p = 0.002); more than one-third of both male and female participants had given or received gifts or money in exchange for sex. Six percent of women and 2% of men reported to have had a disease which they got through sexual contact during the past year (p = 0.036). Nearly one in four women (24%) responded affirmatively when asked if their husband/partner ever slapped them, kicked, dragged or beat them up, or physically forced them to have sexual intercourse.

**Table 2:** Characteristics of study participants, N = 486.

	Total	Males	Females	
Number of participants (%)	486	161 (33.1%)	325 (66.9%)	
	Mean (sd) or percent	Mean (sd) or percent	Mean (sd) or percent	p-value 1
***Sociodemographic characteristics***				
Age (years)	27.4 (8.1)	24.7 (7.3)	28.7 (8.1)	<0.001
Marital status				
Married	46.7	21.1	59.4	<0.001
Never married	46.5	77.0	31.4	
Divorced/separated	6.0	1.9	8.0	
Widowed	0.8	0.0	1.2	
Education				
None	0.4	0.0	0.6	<0.001
Standard 1–6	3.9	1.2	5.2	
Standard 7	50.0	31.7	59.1	
Form 1 or higher	45.7	67.1	35.1	
Employment types				
Student	19.8	39.1	10.2	<0.001
Unemployed	2.9	3.7	2.5	
Self-employed	32.3	27.3	34.8	
Casual laborer	5.3	6.2	4.9	
Employed	20.6	23.6	19.1	
Housewife	19.1	0.0	28.6	
Household composition				
# of adults ages 18–49	1.9 (1.1)	1.8 (1.4)	2.0 (0.9)	0.185
# of children <18	1.5 (1.5)	0.9 (1.4)	1.8 (1.4)	<0.001
***HIV risk factors***				
Lifetime				
# of sexual partners, lifetime	2.3 (2.6)	2.8 (3.8)	2.0 (1.7)	0.002
Ever exchanged gifts or money for sex	38.9	34.2	41.2	0.132
Past year				
# of sexual partners, past year	0.9 (1.5)	1.0 (2.5)	0.9 (0.5)	0.290
Any alcohol consumption	28.6	32.9	26.5	0.138
Travelled and slept away from home	60.9	72.0	55.4	<0.001
Any sexually transmitted disease	4.7	1.9	6.2	0.036
Current exposure to domestic violence			23.7	-
***HIV testing history***				
# of prior HIV tests				
0	24.5	43.5	15.1	<0.001
1–2	34.4	32.9	35.1	
3–4	25.5	17.4	29.5	
5+	15.6	6.2	20.3	
Time of most recent HIV test				
Within the past 1 year	31.5	19.9	37.2	<0.001
Within the past 5 years	68.3	49.7	77.5	<0.001
Self-reported HIV diagnosis 2	0.8	0.6	0.9	1.000

1 Statistical significance was assessed using Student's *t* tests (continuous variables), Fisher's exact test (self-reported HIV diagnosis), and chi-squared tests (all other variables).

2 Four individuals with self-reported HIV diagnosis were excluded from analyses of HIV testing preferences.

The percentage of participants who had tested for HIV at least once was significantly higher among women than men (85% vs. 57%; p<0.001); one in five men (22%) and 37% of women had tested in the past year (p<0.001). While HIV infection was not an exclusion criterion for the study, four participants (0.8%) who reported to be HIV-infected were excluded from analyses of testing preferences, as their preferences likely differ systematically from those who are not infected and those who do not know if they are infected with HIV.

### Direct assessment of preferences

**Table 1** shows the results of the “direct assessment” of preferences, which asked participants to indicate their most and least preferred levels for each attribute, without considering other attributes. While the majority of participants (53%) indicated that they would prefer HIV counseling and testing at their home if the option were available, for one in five participants (22%) home-based counseling and testing was the least preferred alternative. Similarly, while one-third of participants preferred no one to know that they tested, nearly half of the participants preferred their spouse to know, and one in five preferred many people knowing that they tested. More than two-thirds (70%) of participants considered oral swabs the least preferred method for obtaining the sample for the HIV test. A similar share preferred to test at a site where HIV medications would be available should they test HIV positive.

### Results of the discrete choice experiment

Eight persons (0.8%) failed the comprehension test and were excluded from the analysis of DCE data. Additional validity checks assessed literacy and visual acuity, response biases, dominance, and the presence of interactions (**Appendix S2**).

**Table 3** shows the results of gender-specific mixed logit models of participants' stated choices across 8,532 scenarios (474 participants * 9 choice tasks * 2 alternatives; see experimental design/survey administration). Two conclusions can be derived from these results. First, the *estimated mean preference* parameters indicate the relative likelihood of choosing a test with a given attribute-level combination (e.g., “*distance - 1 km from home*”), holding all other factors constant. A larger value indicates a greater likelihood of choosing a test with the specific feature. The rank-ordering of all attribute-level combinations thus describes their relative importance to participants and indicates the most and least important features of testing evaluated in our survey. Among males, “*distance - 1 km*” was the most preferred of all levels of all attributes (estimate  = 1.16), and “*distance - 20 km*” was least preferred (estimate  = -2.30). Among females, “*distance - home*” was the most preferred feature (estimate  = 0.81) and “*distance - 20 km*” was the least preferred (estimate  = −1.47). In between these respective extremes, the importance of other attribute-level combinations is described by their relative position within these numeric ranges. The p-values describe the statistical significance of the association between each feature and participants' preferences.

**Table 3:** Results of gender-specific mixed logit models of discrete choice experiment data on HIV testing preferences in Moshi, Tanzania, 2012–2013 (N = 474) 1.

		Males (N = 159)						Females (N = 315)					
Attribute	Level	Estimated mean preference	(SE)		Estimated standard deviation	(SE)		Estimated mean preference	(SE)		Estimated standard deviation	(SE)	
Distance	Home	0.82	(0.16)	***	0.15	(0.20)		0.81	(0.12)	***	1.29	(0.14)	***
	1 km	1.16	(0.20)	***	0.92	(0.27)	***	0.52	(0.10)	***	0.74	(0.17)	***
	5 km	0.32	(0.15)	*	0.29	(0.21)		0.14	(0.08)		0.60	(0.12)	***
	20 km	−2.30	(0.26)	***	1.36	(0.50)	**	−1.47	(0.15)	***	2.63	(0.30)	***
Confidentiality	Many people	−0.46	(0.14)	**	0.51	(0.23)	*	−0.57	(0.10)	***	1.19	(0.15)	***
	Spouse	0.55	(0.13)	***	1.07	(0.19)	***	0.40	(0.07)	***	0.52	(0.09)	***
	No-one	−0.08	(0.14)		1.58	(0.27)	***	0.16	(0.08)	*	0.67	(0.11)	***
Testing days	Weekdays	−0.17	(0.10)		0.98	(0.15)	***	0.13	(0.05)	*	0.50	(0.08)	***
	Weekends	0.17	(0.10)		0.98	(0.15)	***	−0.13	(0.05)	*	0.50	(0.08)	***
Type of sample	Arm	0.19	(0.14)		2.39	(0.27)	***	0.40	(0.09)	***	1.09	(0.12)	***
	Finger	0.70	(0.16)	***	1.82	(0.26)	***	0.59	(0.09)	***	1.03	(0.13)	***
	Mouth	−0.89	(0.18)	***	4.21	(0.50)	***	−0.99	(0.10)	***	2.12	(0.21)	***
Services if HIV positive	Referral	−0.31	(0.11)	**	1.28	(0.15)	***	−0.34	(0.06)	***	0.69	(0.08)	***
	Medications	0.31	(0.11)	**	1.28	(0.15)	***	0.34	(0.06)	***	0.69	(0.08)	***
*Log likelihood*		*−780.14*						*−1581.67*					
*Number of observations*		*2,862*						*5,670*					

1 Estimates from gender-specific mixed logit models with correlated random coefficients for all attribute levels. Four respondents who reported to be HIV infected and eight respondents who failed the comprehension test were excluded from analyses of testing preferences.

# omitted attribute level; estimates derived using Wald tests.

*, **, and *** indicate statistical significance at the 0.05, 0.01, and 0.001 levels, respectively.

Second, the degree to which respondent preferences were heterogeneous, i.e. the extent to which preferences vary in the sample, is described by the *estimated standard deviation* around each mean preference estimate. Larger values indicate more variation across participants than do smaller ones. For example, from the results it can be inferred that among males “*type of sample – mouth*” had the greatest preference heterogeneity (estimated standard deviation  = 4.21). Evidently, even though on average most respondents did not favor oral HIV testing (estimated mean preference  = −0.89), a few respondents preferred this feature a great deal. Thus, these results describe both the mean preferences for all attribute-level combinations in comparison with one another, and the heterogeneity of preferences among participants.

There were significant differences in the estimated preferences between different attribute-level combinations (not shown). Both male and female participants actually preferred their spouses to know about their HIV test, compared to no one knowing (p = 0.007 for males and p = 0.029 for females) or many people knowing (both p<0.001) about the test. On average, both male and female participants preferred finger pricks or venipuncture to oral testing (all p<0.001), with males preferring finger pricks over venipuncture (p = 0.038).

Testing days (weekdays vs. weekends) and medication availability at the testing site were less important to participants than distance, confidentiality, and method for obtaining the sample, as indicated by the smaller magnitude of the mean preference estimates. Females, on average, had a slight preference for weekday testing (estimate = 0.13; p = 0.015); testing days were not associated with preferences among males (estimate = 0.17; p = 0.092). Both male (estimate = 0.31; p = 0.007) and female (estimate = 0.34; p<0.001) participants preferred testing in places where medications are available, as opposed to places providing referrals for those who test HIV positive. Large standard deviations on each of these parameters suggest that, while the effects are small on average, some people have strong preferences for each attribute level evaluated.

### Variation in preferences across subpopulations

**Figure 3:**
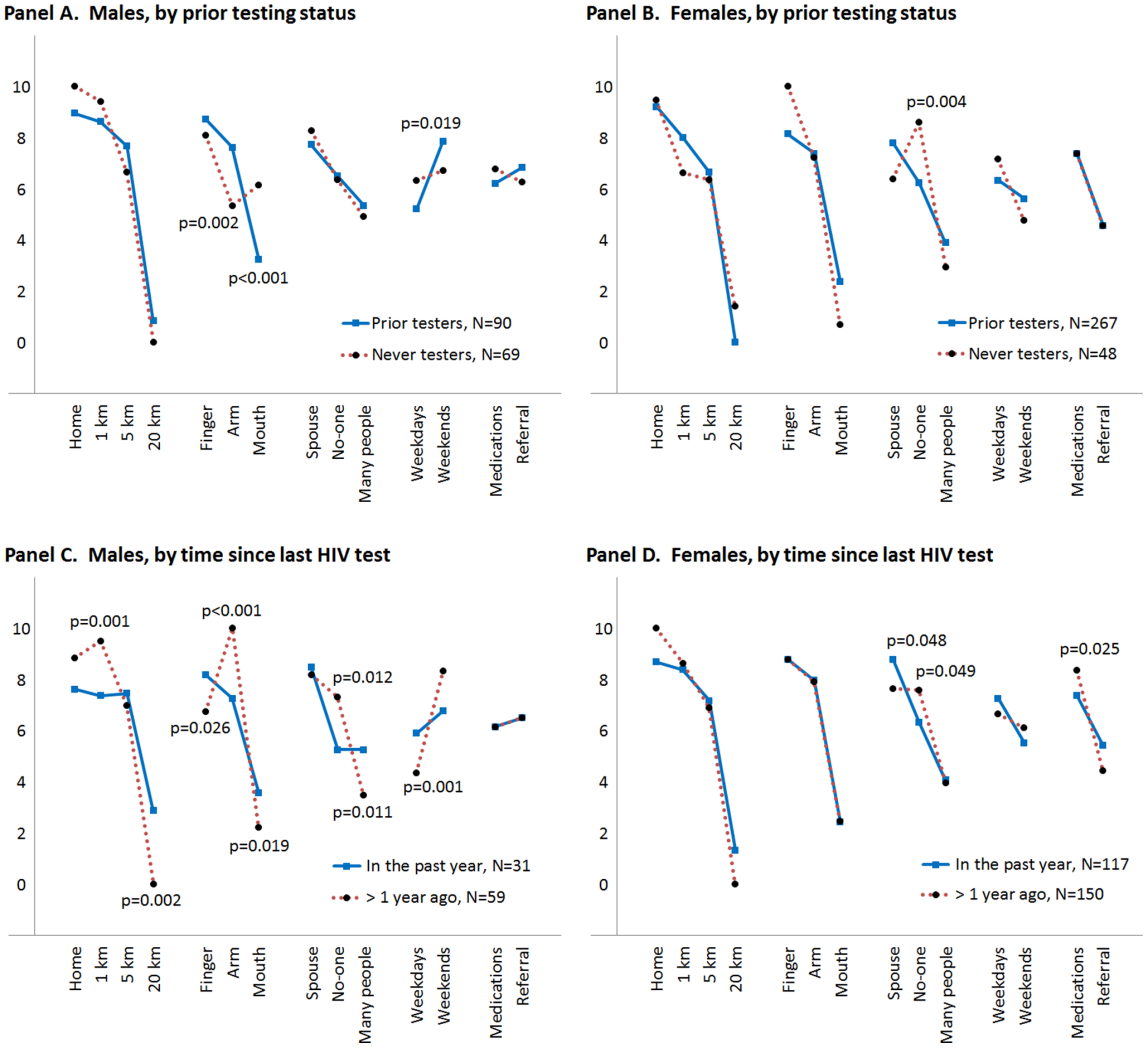
Scaled estimates of HIV testing preferences by gender, prior HIV testing status and time since the last HIV test. Gender-specific estimates of the effect of each attribute level on HIV testing preferences, separately for prior testers vs. those who never tested (Panels A and B), and those who tested in the past year vs. those who tested more than 1 year ago (Panels C and D). Models included correlated random main effects and fixed interactions between attribute levels and participants' HIV testing histories. p-values indicate statistically significant differences between the respective groups, as measured by the interaction terms. Coefficients were re-scaled to range from 0 to 10.

To explore whether the heterogeneity observed in Table 3 is explained by differences between those who previously tested for HIV and those who never tested, we estimated additional gender-specific mixed logit models that included interactions between attribute levels and (1) HIV testing status (**Figure 3**, Panels A and B), and (2) time since the most recent HIV test among participants who previously tested (Panels C and D).

Compared with those who had tested previously, males who had never tested were more likely to prefer oral HIV tests (p<0.001) and less likely to prefer venipuncture (p = 0.002; Panel A). The compressed distribution of the estimated distance parameters among males who had tested, especially those who tested in the past year (Panel C), suggests that distance may be more important to those who do not regularly test for HIV. Males who tested more than one year ago were more likely to prefer venipuncture (p<0.001), weekend testing (p = 0.001), and that no one know they tested (p = 0.012).

Among females, those who had never tested for HIV preferred that no one know that they tested (p = 0.004; Panel B). Females who tested more than one year ago were less likely to prefer their spouse knowing about the HIV test (p = 0.048) and had a stronger preference for medication availability at the testing site (p = 0.025; Panel D).

Wald tests of whether the interaction parameters for prior HIV testing status and the time since the last HIV test jointly improve the fit of the respective models suggest significant improvements for males (p = 0.007 and p = 0.0053, respectively), but not for females (p = 0.1421 and 0.1121, respectively).

## Discussion

We used DCE methodology to evaluate preferences for HIV testing in a rigorously selected population-based sample in Moshi, Tanzania. All attributes of HIV testing options evaluated in the survey – distance to testing, confidentiality, method for obtaining the sample, weekday vs. weekend testing, and availability of HIV medications at the testing site – were statistically significantly associated with population preferences for testing.

Among the five attributes and across the levels evaluated, the strongest preferences were observed on the distance attribute, with testing closer to home preferred to testing farther from home. This finding supports policy initiatives to improve the geographic accessibility of testing services. However, while home-based testing was preferred on average, both direct assessments and the DCE results suggest that it is not universally preferred. In the direct assessment, one-fifth of the participants cited home testing as their least preferred option; in the DCE, a large standard deviation of the parameter estimate for home-based testing, particularly among females, suggests that a sizeable proportion of the sample derived a negative utility from testing at home. These findings stand in contrast to reported high rates of acceptability of multiple large home-based testing campaigns [15]. If the universal, community-based nature of such campaigns was critical to their success, then comparable rates may not be achieved when the offer of home-based testing is directed at individual persons or households.

Preferences related to the confidentiality of testing may signal an important shift in individual motives for HIV testing. In direct assessments, only one-third of participants preferred that no one know that they tested. Somewhat surprisingly, half of respondents actually preferred that their spouse know that they tested. Qualitative inquiries during the pre-testing phase of this study indicated that with HIV prevalence declining [45], an increasing number of individuals may test for HIV to demonstrate their HIV-seronegative status to their partner(s). Broad shifts in the underlying motivation for testing have the potential to destigmatize testing as a marker of risk, thereby improving acceptability and uptake. Yet, if a resulting increase in testing rates is concentrated in low-risk populations, population-based HIV testing efforts will become less cost effective [62].

The third attribute strongly associated with preferences is the method for obtaining the sample for the HIV test. Some participants preferred finger pricks and others venipuncture, whereas many were averse to oral swabs. Qualitative assessments during pre-testing suggest that participants' lack of familiarity with oral swabs for HIV testing and a lack of confidence in their accuracy were the primary reasons for their stated preferences. Some individuals believed that only a venipuncture provides sufficient blood volume for an accurate HIV test. Without appropriate educational campaigns about the comparable accuracy of different tests, it is unlikely that benefits such as reduced invasiveness or pain will broadly improve uptake of HIV testing in the study area. This finding is particularly relevant given the potential increased availability of oral HIV test kits in Tanzania in the near future and may inform the debate about the acceptability and benefits of other options such as self-testing [63].

Contrary to our expectations, medication availability at the testing sites and the availability of HIV testing on weekends, while significant, were not as important to participants as distance, confidentiality, and the method for obtaining the sample. This observation highlights the added value of DCE-based methods over conventional opinion surveys. When respondents are not asked to make tradeoffs, many characteristics are considered important, especially among populations where social desirability may bias responses [64]. DCEs allow for the prioritization of different attributes and can guide the optimal allocation of limited resources.

The results point to intervention options for increasing uptake of HIV testing in specific populations. Males who never tested for HIV had a greater aversion to venipuncture than those who had previously tested, and those who had not tested in the past year had a strong preference for testing on weekends. Offering clients a choice between different methods for obtaining the testing sample and increasing the availability of weekend testing may increase uptake of testing among males. To our knowledge, weekend testing is routinely available at only one facility in the study area. Among females, confidentiality concerns continue to be a barrier to testing, highlighting the need to reinforce confidentiality training among HIV counselors and to ensure that the environment at testing sites promotes privacy.

We acknowledge several limitations of the study. HIV testing options were described with only five attributes. Four additional attributes were evaluated in our pilot study, including waiting time, counseling time, having the option to choose a counselor, and testing at an HIV-specific vs. general-service facility. Other attributes were considered during formative work but determined to be of lower importance. The five attributes were selected to minimize biases from omitted variables and ensure full comprehension by participants. Second, while this study demonstrated associations between testing characteristics and participants' preferences, no predictions can be made about uptake of specific testing options, because the DCE did not include a “no test” alternative, and no inferences can be made about actual testing behaviors. Finally, the results indicate significant heterogeneity in HIV testing preferences. While we highlighted variation in preferences by gender and HIV testing history, other characteristics such as HIV-related stigma, socio-economic characteristics, and positive or negative experiences with prior HIV tests may contribute to variation in preferences. Variation in testing preferences across these factors has implications for the optimal combination of population-based testing options and should be analyzed in future research.

This study is the first to use DCE methodology to assess HIV testing preferences in sub-Saharan Africa. Using a population-based sample, we identify distinct preference patterns across various subpopulations, including males and females and those who previously tested for HIV and those who have never tested. DCEs are a useful tool for exploring HIV testing preferences, highlight the limitations of conventional survey methods for assessing preferences in populations prone to social desirability bias, and can inform the design of targeted HIV testing interventions.

## Supporting Information

Appendix S1 **Survey instrument for the paper-based supplemental survey for the “** ***HIV Testing Preferences in Tanzania*** **” (TP-TZ) study.**
(PDF)

Appendix S2 **Validity checks.**
(DOC)
