# Supplementary material for: Heterogeneous HIV Testing Preferences in an Urban Setting in Tanzania: Results from a Discrete Choice Experiment
Source: PLoS One. 2014 Mar 18;9(3):e92100. doi: 10.1371/journal.pone.0092100 (PMC3958474; doi:10.1371/journal.pone.0092100)
Supplement: Appendix S1 — Survey instrument for the paper-based supplemental survey for the “ HIV Testing Preferences in Tanzania ” (TP-TZ) study. (PDF) [file pone.0092100.s001.pdf]

HCT-DCE v50, created 10 Sep 2012 ▶ Tracking #: __ __ __ __

Survey facepage for

DCE I - English

8 section(s):

1. **Attitudes** [p. 2]
2. **HIV Testing** [p. 6]
3. **HIV Risk** [p. 8]
4. **Knowledge & Stigma** [p. 9]
5. **Relationship** [p. 10]
6. **Health** [p. 11]
7. **Sociodemographics** [p. 12]
8. **Socioeconomics** [p. 13]

Fill at time of data collection:

Interviewer: ______________________________

Participant ID: ___ ___ - ___ ___ ___ - ___

Interview #: _________

Date of data collection: (YYYY / MM / DD)

___ ___ ___ ___ / ___ ___ / ___ ___

Fill before data entry:

Tracking #: ___ ___ ___ ___

Confirmation #: ___ ___ ___ - ___ ___ ___ - ___

Status (check when complete):

___ **Quality assurance**

___ **Data entry**

___ **Scanned**

Notes:

ef 02 9f 81 b8

Begin on next page ➔

DCE I
Facepage
Page **1** of **13**
ef029f81b850d61fd9c40c8040fff17f522d9402

Ostermann et al. - Heterogeneous HIV testing preferences in an urban setting in Tanzania: Results from a Discrete Choice Experiment

HCT-DCE v50, created 10 Sep 2012 ▶ Tracking #: __ __ __ __

q:4j3 **Supplemental Survey**

1) q:4io Participant ID

____

Or: DK / RF / MI [strike across for NA]

2) q:4ip DCE completed on …

Mark one:

- ❍ a) Paper
- ❍ b) iPad

Or: DK / RF / MI [strike across for NA]

3) q:4iq Interview ID:

____

Or: DK / RF / MI [strike across for NA]

q:4h4 **Attitudes**

☞ q:4h6 Now we are going to ask you questions about HIV testing in and around Moshi. Even if you don't think you would ever test for HIV, we would like you to consider the following questions as if you actually decided to get tested.

4) q:4f6 Do you know of a place where people can go to get tested for the AIDS virus?

Mark one:

- ❍ a) Yes
- ❍ b) No

Or: DK / RF / MI [strike across for NA]

5) q:4h8 IF YES:
Please tell me all of the places in Moshi that you know of that offer HIV testing.

Mark all that apply (do NOT read to respondent):

- ❒ a) Angaza
- ❒ b) C.C.P.
- ❒ c) KCMC
- ❒ d) Kiboroloni
- ❒ e) Kilema
- ❒ f) Kiusa
- ❒ g) Kiwakkuki
- ❒ h) Majengo
- ❒ i) Mawenzi
- ❒ j) Pasua
- ❒ k) Rainbow
- ❒ l) Siima
- ❒ m) Other ________________

Or: DK / RF / MI [strike across for NA]

6) q:4g7 Now I will read to you a list of places in Moshi that offer HIV testing. Of these places, where would you prefer to test?

Mark one (READ responses aloud):

- ❍ a) KCMC
- ❍ b) Mawenzi Hospital
- ❍ c) Kiwakkuki
- ❍ d) Angaza
- ❍ e) Kiusa
- ❍ f) Rainbow Centre
- ❍ g) C.C.P.
- ❍ h) Kiboroloni Dispensary
- ❍ i) Majengo Health Centre
- ❍ j) Pasua Health Centre
- ❍ k) Siima
- ❍ l) Other ________________

Or: DK / RF / MI [strike across for NA]

➥ q:4i1 ▪ ▢ If clients states he/she will NEVER TEST, mark NA and skip to **#10, p. 3** (q:4ii)

7) q:4g8 You said you would prefer to test at […].
Have you previously tested there for HIV?

Mark one:

- ❍ a) Yes
- ❍ b) No

Or: DK / RF / MI [strike across for NA]

8) q:4g9 Why would you prefer to test at this place?

____

Or: DK / RF / MI [strike across for NA]

9) q:4h9 INTERVIEWER RECODE.

Mark all that apply (do NOT read to respondent):

- ❒ a) Anonymity
- ❒ b) Closest facility
- ❒ c) Accuracy / confidence in the result
- ❒ d) Counselor characteristics
- ❒ e) Medications available
- ❒ f) Other services provided
- ❒ g) Testing times
- ❒ h) Other

Or: DK / RF / MI [strike across for NA]

Continue on next page ➔

ef029f81b850d61fd9c40c8040fff17f522d9402

10) q:4ii
If testing was offered at home, and a counselor from […preferred place] came to your home for counseling and testing, would you prefer to test …

Mark one:
❍ a) At your home
❍ b) [... at the preferred testing place]
Or: DK / RF / MI [strike across for NA]

➡ q:4hb
- ▢ If clients states he/she will NEVER TEST, mark NA and skip to **#25, p. 4** (q:4gw)

11) q:4j4
On which day of the week, Monday through Sunday, would you prefer to test?

Mark one (do NOT read to respondent):
❍ a) Monday
❍ b) Tuesday
❍ c) Wednesday
❍ d) Thursday
❍ e) Friday
❍ f) Saturday
❍ g) Sunday
❍ h) Not important
Or: DK / RF / MI [strike across for NA]

12) q:4j5
At what time of the day would you prefer to test?

Mark one (do NOT read to respondent):
❍ a) 6 - 8am
❍ b) 8 - 10am
❍ c) 10am - 4pm
❍ d) 4 - 6pm
❍ e) 6 - 8pm
❍ f) 8pm - 6am
❍ g) Not important
Or: DK / RF / MI [strike across for NA]

13) q:4gc
Would you prefer …

Mark one:
❍ a) to make an appointment before going for testing
❍ b) to just go there
Or: DK / RF / MI [strike across for NA]

14) q:4gb
Would you prefer …

Mark one:
❍ a) to have someone escort you to go for HIV testing
❍ b) go for HIV testing on your own
Or: DK / RF / MI [strike across for NA]

15) q:4gn
Would you prefer to test …

Mark one:
❍ a) At a place where you are asked for your name
❍ b) At a place where you do not have to give your name?
❍ c) Not important
Or: DK / RF / MI [strike across for NA]

16) q:4gq
How much time do you think it takes to test for HIV, from the moment you enter the testing place until you are finished?

____

Or: DK / RF / MI [strike across for NA]

17) q:4gi
Would you prefer …

Mark one:
❍ a) A counselor who is older than you
❍ b) A counselor who is younger than you
❍ c) A counselor who is the same age as you
❍ d) Not important
Or: DK / RF / MI [strike across for NA]

18) q:4gj
Would you prefer …

Mark one:
❍ a) A male counselor
❍ b) A female counselor
❍ c) Not important
Or: DK / RF / MI [strike across for NA]

19) q:4gk
Would you prefer …

Mark one:
❍ a) A counselor who knows you
❍ b) A counselor who doesn't know you
❍ c) Not important
Or: DK / RF / MI [strike across for NA]

20) q:4gl
Would you prefer to be tested for HIV by …

Mark one:
❍ a) A doctor
❍ b) A nurse
❍ c) An HIV counselor
❍ d) Not important
Or: DK / RF / MI [strike across for NA]

Continue on next page ➔

ef029f81b850d61fd9c40c8040fff17f522d9402

21) q:4gm
Let's say you have already tested and you are going to test again. Would you prefer …

Mark one:
- ❍ a) The same counselor who counseled you last time
- ❍ b) A different counselor
- ❍ c) Not important

Or: DK / RF / MI [strike across for NA]

22) q:4gg
Would you prefer to get tested for HIV alone or together with someone else?

Mark one:
- ❍ a) Alone
- ❍ b) With someone else

Or: DK / RF / MI [strike across for NA]

23) q:4gh
IF 'WITH SOMEONE ELSE':
With whom would you prefer to test?

Mark one (do NOT read to respondent):
- ❍ a) Spouse
- ❍ b) Sexual partner
- ❍ c) Child(ren)
- ❍ d) Other family member(s)
- ❍ e) Entire family
- ❍ f) Friend
- ❍ g) Other ______________________ ✍

Or: DK / RF / MI [strike across for NA]

24) q:4gs
Would you prefer to receive the result from the counselor right away, or to get a phone call later with the result?

Mark one:
- ❍ a) From the counselor
- ❍ b) Later by phone

Or: DK / RF / MI [strike across for NA]

25) q:4gw
In your opinion, how accurate are HIV tests [in your preferred testing site]?

Mark one (READ responses aloud):
- ❍ a) Completely accurate
- ❍ b) Mostly accurate
- ❍ c) Inaccurate

Or: DK / RF / MI [strike across for NA]

26) q:4it
Do you think there are differences in the accuracy of the test between HIV testing centres?

Mark one (READ responses aloud):
- ❍ a) You think yes
- ❍ b) You think no

Or: DK / RF / MI [strike across for NA]

27) q:4gv
If you found out that you are HIV positive, which of the following would you prefer?

Mark one (READ responses aloud):
- ❍ a) You inform your partner that you have HIV.
- ❍ b) The HIV testing center assists you with informing your partner.
- ❍ c) You prefer not to inform your partner.

Or: DK / RF / MI [strike across for NA]

28) q:4gt
If you test for HIV and don't tell anyone, how likely is it that some other person will find out that you tested?

Mark one (READ responses aloud):
- ❍ a) Very likely
- ❍ b) Somewhat likely
- ❍ c) Not likely

Or: DK / RF / MI [strike across for NA]

29) q:4gy
If an oral HIV test was offered in Tanzania, would you be …

Mark one:
- ❍ a) willing to test
- ❍ b) not willing to test

Or: DK / RF / MI [strike across for NA]

30) q:4gz
Like persons with diabetes who can test themselves, if a self-test was available, where you can test for HIV yourself and get the results in the privacy of your own home, would you be …

Mark one:
- ❍ a) willing to test
- ❍ b) not willing to test

Or: DK / RF / MI [strike across for NA]

31) q:4h0
When, if ever, do you think you will test for HIV [next time]?

Mark one (READ responses aloud):
- ❍ a) Within the next 3 months
- ❍ b) Within the next 1 year
- ❍ c) More than 1 year from now
- ❍ d) Never

Or: DK / RF / MI [strike across for NA]

➥ q:4ib
- ▢ If NEVER, skip to **#34, p. 5** (q:4h3)

Continue on next page ➔

HCT-DCE v50, created 10 Sep 2012 ▶ Tracking #: __ __ __ __

32) q:4i4 Would you prefer to receive HIV counseling and testing when you go to see a doctor for other reasons, or would you prefer to just test for HIV?

Mark one:

❍ a) While seeing a doctor for other reasons

❍ b) HIV testing only

Or: DK / RF / MI [strike across for NA]

33) q:4gd Would you prefer to receive HIV counseling and testing when you get tested for Malaria or would you prefer to just test for HIV?

Mark one:

❍ a) While testing for malaria

❍ b) HIV testing only

Or: DK / RF / MI [strike across for NA]

34) q:4h3 If you had to choose, what would you consider most important in deciding where to test for HIV?

Mark one (READ responses aloud):

❍ a) Privacy and confidentiality

❍ b) Accuracy of the test

❍ c) Access to testing and convenience

Or: DK / RF / MI [strike across for NA]

☞ q:4iy You said it takes time to test, and testing may interfere with your normal activities. If someone asked you to change your plans for tomorrow in order to test for HIV, it may be best if they compensate you for your time.

35) q:4hw If someone were to offer you TSH 5,000 to test for HIV tomorrow, would you test?

Mark one:

❍ a) Yes

❍ b) No

Or: DK / RF / MI [strike across for NA]

➡ q:4i0

- ▢ If NO skip to **#37, p. 5** (q:4hy)

36) q:4hx If someone were to offer you TSH 2,000 to test for HIV tomorrow, would you test?

Mark one:

❍ a) Yes

❍ b) No

Or: DK / RF / MI [strike across for NA]

➡ q:4hz

- ▢ If YES, skip to **"HIV Testing", p. 6** (q:4ex)

37) q:4hy If someone were to offer you TSH 10,000 to test for HIV tomorrow, would you test?

Mark one:

❍ a) Yes

❍ b) No

Or: DK / RF / MI [strike across for NA]

➡ q:4hz

- ▢ If YES, skip to **"HIV Testing", p. 6** (q:4ex)

38) q:4h1 If NO:
How much would they have to offer so that you would test for HIV tomorrow?

______________________________ ✍

Or: DK / RF / MI [strike across for NA]

Continue on next page ➔

ef029f81b850d61fd9c40c8040fff17f522d9402

q:4ex
HIV Testing

39) q:4ad
In your lifetime, how many times have you been tested for HIV?

____

Or: DK / RF / MI [strike across for NA]

➡ q:4fp
- ☐ If answer is 0 or NEVER, skip to **#54, p. 7** (q:4b0)

40) q:4j8
Have you ever tested at a hospital or health centre?

Mark one:
- ❍ a) Yes
- ❍ b) No

Or: DK / RF / MI [strike across for NA]

41) q:4j9
Have you ever tested at a community VCT centre?

Mark one:
- ❍ a) Yes
- ❍ b) No

Or: DK / RF / MI [strike across for NA]

42) q:4ja
Have you ever tested during mobile VCT?

Mark one:
- ❍ a) Yes
- ❍ b) No

Or: DK / RF / MI [strike across for NA]

43) q:4jb
Have you ever tested anywhere else?

Mark one:
- ❍ a) No
- ❍ b) Other ____

Or: DK / RF / MI [strike across for NA]

44) q:4iv
WOMEN ONLY:
Have you ever tested for HIV as part of PMTCT?

Mark one:
- ❍ a) Yes
- ❍ b) No

Or: DK / RF / MI [strike across for NA]

45) q:4fh
Did you test for HIV during the National Campaign in 2007?

Mark one:
- ❍ a) Yes
- ❍ b) No

Or: DK / RF / MI [strike across for NA]

46) q:4ae
In which year and month did you test the last time?

Year: _____ / Month: _____ / Day: _____
Or: DK / RF / MI [strike across for NA]

47) q:4g4
Was this in Moshi or elsewhere?

Mark one:
- ❍ a) Moshi
- ❍ b) Other ____

Or: DK / RF / MI [strike across for NA]

48) q:4fg
Where did you test?

Mark one (do NOT read to respondent):
- ❍ a) Angaza
- ❍ b) C.C.P.
- ❍ c) KCMC
- ❍ d) Kiboroloni
- ❍ e) Kilema
- ❍ f) Kiusa
- ❍ g) Kiwakkuki
- ❍ h) Majengo
- ❍ i) Mawenzi
- ❍ j) Pasua
- ❍ k) Rainbow
- ❍ l) Siima
- ❍ m) Other ____

Or: DK / RF / MI [strike across for NA]

49) q:4hc
INTERVIEWER RECODE.

Mark one (do NOT read to respondent):
- ❍ a) Dispensary
- ❍ b) Health center
- ❍ c) Hospital
- ❍ d) Community VCT center
- ❍ e) Mobile VCT
- ❍ f) Other ____

Or: DK / RF / MI [strike across for NA]

Continue on next page ➔

HCT-DCE v50, created 10 Sep 2012 ▶ Tracking #: __ __ __ __

50) q:4j6
The last time you tested …

Mark one (READ responses aloud):
- ❍ a) Did you ask for the test yourself?
- ❍ b) Was it required because you were pregnant?
- ❍ c) Was it required for other reasons?
- ❍ d) Was it suggested that you test and you accepted?

Or: DK / RF / MI [strike across for NA]

51) q:4is
What was the test result?

Mark one:
- ❍ a) HIV+
- ❍ b) HIV-

Or: DK / RF / MI [strike across for NA]

52) q:4ag
Have you ever told your husband/wife/partner your test result?

Mark one:
- ❍ a) Yes
- ❍ b) No

Or: DK / RF / MI [strike across for NA]

53) q:4i2
Have you told anyone else your test result?

Mark one:
- ❍ a) Yes
- ❍ b) No

Or: DK / RF / MI [strike across for NA]

54) q:4b0
Has your husband/wife/partner ever tested for HIV?

Mark one:
- ❍ a) Yes
- ❍ b) No
- ❍ c) Has no partner

Or: DK / RF / MI [strike across for NA]

55) q:4ah
IF YES:
What was your husband's/wife's test result?

Mark one:
- ❍ a) HIV+
- ❍ b) HIV-

Or: DK / RF / MI [strike across for NA]

56) q:4id
IF HIV-
Of all the adults in this household, have any of them tested HIV positive?

Mark one:
- ❍ a) Yes
- ❍ b) No

Or: DK / RF / MI [strike across for NA]

57) q:4g0
How many children under the age of 18 live in this household?

____

Or: DK / RF / MI [strike across for NA]

58) q:4i9
Of all the children in this household, how many have tested for HIV?

____

Or: DK / RF / MI [strike across for NA]

59) q:4ic
IF 1 OR MORE:
Have any of these children tested HIV positive?

Mark one:
- ❍ a) Yes
- ❍ b) No

Or: DK / RF / MI [strike across for NA]

➡ q:4ir
- ▢ If respondent has tested skip to **"HIV Risk", p. 8** (q:4fi)

60) q:4ez
What are the reasons you have never tested for HIV?

Mark all that apply (do NOT read to respondent):
- ❒ a) Never thought of getting tested
- ❒ b) Don't think I'm at risk
- ❒ c) Afraid of needle
- ❒ d) Don't have time for testing
- ❒ e) Nervous to get results
- ❒ f) Worried about partner's reaction
- ❒ g) Worried about other people knowing
- ❒ h) Other ________________

Or: DK / RF / MI [strike across for NA]

61) q:4if
What, if anything, would make you get tested for HIV?

____

Or: DK / RF / MI [strike across for NA]

Continue on next page ➔

HCT-DCE v50, created 10 Sep 2012 ▶ Tracking #: __ __ __ __

q:4fi
HIV Risk

62) q:4es
In total, with how many different people have you had sexual intercourse in your lifetime?

____

Or: DK / RF / MI [strike across for NA]

q:4fs
▪ ▢ If NEVER, mark N/A and skip to **#68, p. 8** (q:4ey)

63) q:4er
In total, with how many different people have you had sexual intercourse in the last 12 months?

____

Or: DK / RF / MI [strike across for NA]

64) q:4fa
Of all the sexual partners you have ever had, do you think that any of them have HIV?

Mark one (READ responses aloud):
❍ a) You think yes
❍ b) You think no

Or: DK / RF / MI [strike across for NA]

65) q:4fb
Of all the sexual partners you have ever had, have any of them died?

Mark one:
❍ a) Yes
❍ b) No

Or: DK / RF / MI [strike across for NA]

66) q:4ev
During the last 12 months, have you had a disease which you got through sexual contact?

Mark one:
❍ a) Yes
❍ b) No

Or: DK / RF / MI [strike across for NA]

67) q:4fj
Have you ever given or received gifts or money for sex?

Mark one:
❍ a) Yes
❍ b) No

Or: DK / RF / MI [strike across for NA]

68) q:4ey
Which of these two statements do you agree with more?

Mark one (READ responses aloud):
❍ a) I believe that I am living with HIV
❍ b) I believe that I do not have HIV

Or: DK / RF / MI [strike across for NA]

69) q:4g2
If you were to test for HIV today, what do you think are the chances that you would be found to be HIV infected

Mark one (READ responses aloud):
❍ a) None
❍ b) Almost none
❍ c) Less than half
❍ d) About half
❍ e) More than half

Or: DK / RF / MI [strike across for NA]

Continue on next page ➔

HCT-DCE v50, created 10 Sep 2012 ▶ Tracking #: __ __ __ __

q:4f8 **HIV Knowledge**

70) q:4d8 Can people get the AIDS virus from mosquito bites?

Mark one:
❍ a) Yes
❍ b) No
Or: DK / RF / MI [strike across for NA]

71) q:4d9 Can people reduce their chance of getting the AIDS virus by using a condom every time they have sex?

Mark one:
❍ a) Yes
❍ b) No
Or: DK / RF / MI [strike across for NA]

72) q:4da Can people get the AIDS virus by sharing food with a person who has AIDS?

Mark one:
❍ a) Yes
❍ b) No
Or: DK / RF / MI [strike across for NA]

73) q:4db Can people get the AIDS virus because of witchcraft or other supernatural means?

Mark one:
❍ a) Yes
❍ b) No
Or: DK / RF / MI [strike across for NA]

74) q:4dc Is it possible for a healthy-looking person to have the AIDS virus?

Mark one:
❍ a) Yes
❍ b) No
Or: DK / RF / MI [strike across for NA]

75) q:4hf Is it possible for the long term partner of an HIV positive person to be HIV negative?

Mark one:
❍ a) Yes
❍ b) No
Or: DK / RF / MI [strike across for NA]

76) q:4dd Can the virus that causes AIDS be transmitted from a mother to her baby …

Mark all that apply (read ONE-by-ONE):
❒ a) during pregnancy?
❒ b) during delivery?
❒ c) by breastfeeding?
❒ d) None of the above
Or: DK / RF / MI [strike across for NA]

77) q:4de Are there any special drugs that a doctor or a nurse can give to a woman infected with the AIDS virus to reduce the risk of transmission to the baby?

Mark one:
❍ a) Yes
❍ b) No
Or: DK / RF / MI [strike across for NA]

78) q:4d3 Would you buy fresh vegetables from a shopkeeper or vendor if you knew that this person had the AIDS virus, but if s/he was not sick?

Mark one:
❍ a) Yes
❍ b) No
Or: DK / RF / MI [strike across for NA]

79) q:4d4 If a member of your family got infected with the AIDS virus, would you want it to remain a secret or not?

Mark one:
❍ a) Yes, remain a secret
❍ b) No
Or: DK / RF / MI [strike across for NA]

80) q:4d5 If a member of your family became sick with AIDS, would you be willing to care for her or him in your own household?

Mark one:
❍ a) Yes
❍ b) No
Or: DK / RF / MI [strike across for NA]

81) q:4d6 In your opinion, if a female teacher has the AIDS virus but is not sick, should she be allowed to continue teaching in the school?

Mark one:
❍ a) Should be allowed
❍ b) Should not be allowed
Or: DK / RF / MI [strike across for NA]

Continue on next page ➔

HCT-DCE v50, created 10 Sep 2012 ▶ Tracking #: __ __ __ __

q:4fk **Interviewer: Women only.**

Relationship

☞ q:4i5 Now I will ask you some sensitive questions. I want to remind you that your answers will remain confidential and you can refuse to answer any questions.

82) q:4f3 Are you pregnant?

Mark one:

❍ a) Yes

❍ b) No

Or: DK / RF / MI [strike across for NA]

83) q:4dh Can you say no to your husband/partner if you do not want to have sexual intercourse?

Mark one:

❍ a) Yes

❍ b) No

Or: DK / RF / MI [strike across for NA]

84) q:4di Could you ask your husband/partner to use a condom if you wanted him to?

Mark one:

❍ a) Yes

❍ b) No

Or: DK / RF / MI [strike across for NA]

85) q:4dk Does your husband/partner ever …

Mark all that apply (read ONE-by-ONE):

❒ a) slap you?

❒ b) kick you, drag you or beat you up?

❒ c) physically force you to have sexual intercourse with him even when you did not want to?

❒ d) None of the above

Or: DK / RF / MI [strike across for NA]

➡ q:4ft

- ▢ If c) is YES, skip next question.

86) q:4do At any time in your life, as a child or as an adult, has anyone ever forced you in any way to have sexual intercourse or perform any other sexual acts?

Mark one:

❍ a) Yes

❍ b) No

Or: DK / RF / MI [strike across for NA]

⚠ q:4i6 IF YES to either of the last 2 questions, inform the client that she may contact: KWIECO at 027 275 1121.

☞ q:4iz If you encounter problems like those you mentioned, you may seek help or counseling from KWIECO, an organization that helps with issues related to gender violence. The organization is located in the Lucas Construction Building, next to MDC, Arusha Road, in Moshi Town.

Continue on next page ➔

q:4ew
Health

87) q:4aw
In general, would you say your health is:

Mark one (READ responses aloud):
- ❍ a) Excellent
- ❍ b) Very good
- ❍ c) Good
- ❍ d) Fair
- ❍ e) Poor

Or: DK / RF / MI [strike across for NA]

88) q:4i3
When was the last time you visited a health facility for care for yourself?

Mark one:
- ❍ a) In the last 12 months
- ❍ b) 1-2 years ago
- ❍ c) 3-5 years ago
- ❍ d) More than 5 years ago

Or: DK / RF / MI [strike across for NA]

89) q:4g6
Were you offered HIV testing?

Mark one:
- ❍ a) Yes
- ❍ b) No

Or: DK / RF / MI [strike across for NA]

90) q:4e2
Did you give a blood sample?

Mark one:
- ❍ a) Yes
- ❍ b) No

Or: DK / RF / MI [strike across for NA]

91) q:4fu
Within the last 3 months, have you had any of the following?

Mark all that apply (read ONE-by-ONE):
- ❒ a) Fever
- ❒ b) Cough
- ❒ c) Coughing up blood
- ❒ d) Sweats at night soaking the sheets
- ❒ e) Diarrhea
- ❒ f) Genital ulcers/discharge
- ❒ g) Rash
- ❒ h) None of the above

Or: DK / RF / MI [strike across for NA]

92) q:4fe
Have you ever been treated for tuberculosis?

Mark one:
- ❍ a) Yes
- ❍ b) No

Or: DK / RF / MI [strike across for NA]

93) q:4fv
How often did you have a drink containing alcohol in the past year?

Mark one (READ responses aloud):
- ❍ a) Never
- ❍ b) Monthly or less
- ❍ c) 2-4 times per month
- ❍ d) 2-3 times per week
- ❍ e) 4 or more times per week

Or: DK / RF / MI [strike across for NA]

➡ ▪ ▢ If NEVER, skip to next section.

q:4fy

94) q:4fw
How many drinks did you have on a typical day when you were drinking in the past year?

Mark one (READ responses aloud):
- ❍ a) 1 or 2
- ❍ b) 3 or 4
- ❍ c) 5 or 6
- ❍ d) 7 to 9
- ❍ e) 10 or more

Or: DK / RF / MI [strike across for NA]

95) q:4fx
How often did you have six or more drinks on one occasion during the past year?

Mark one (READ responses aloud):
- ❍ a) Never
- ❍ b) Less than monthly
- ❍ c) Monthly
- ❍ d) Weekly
- ❍ e) Daily or almost daily

Or: DK / RF / MI [strike across for NA]

Continue on next page ➔

ef029f81b850d61fd9c40c8040fff17f522d9402

HCT-DCE v50, created 10 Sep 2012 ▶ Tracking #: __ __ __ __

q:4fm
Demographics

☞ Now I will finish the survey by asking you some simple questions that are not related to HIV or HIV testing.

q:4ig

96) q:4aa
Gender

Mark one:
- ❍ a) Male
- ❍ b) Female

Or: DK / RF / MI [strike across for NA]

97) q:4dy
How old were you at your last birthday?

______

Or: DK / RF / MI [strike across for NA]

98) q:4dp
What is your relationship to the head of the household?

Mark one:
- ❍ a) Head of household
- ❍ b) Wife / husband / partner
- ❍ c) Son or daughter
- ❍ d) Son-in-law or daughter-in-law
- ❍ e) Brother or sister
- ❍ f) Other relative
- ❍ g) Not related

Or: DK / RF / MI [strike across for NA]

99) q:4fd
How long have you lived in Moshi?

Years: _____ / Months: _____

Or: DK / RF / MI [strike across for NA]

100) q:4ha
In the last 12 months, have you traveled away from your home community and slept away?

Mark one:
- ❍ a) Yes
- ❍ b) No

Or: DK / RF / MI [strike across for NA]

101) q:4ai
Which of the following best describes your current employment status?

Mark one (READ responses aloud):
- ❍ a) Student
- ❍ b) Unemployed
- ❍ c) Self-employed
- ❍ d) Casual laborer
- ❍ e) Employed
- ❍ f) Housewife
- ❍ g) Other ______

Or: DK / RF / MI [strike across for NA]

102) q:4ay
What is the highest level of education you have completed?

Mark one:
- ❍ a) None
- ❍ b) Standard 1-6
- ❍ c) Standard 7
- ❍ d) Form 1-4
- ❍ e) Form 5 or higher

Or: DK / RF / MI [strike across for NA]

103) q:4j7
Are you married?

Mark one:
- ❍ a) Yes
- ❍ b) No

Or: DK / RF / MI [strike across for NA]

104) q:4ac
IF NO:
What is your marital status?

Mark one (READ responses aloud):
- ❍ a) Widowed
- ❍ b) Divorced / separated
- ❍ c) Never married

Or: DK / RF / MI [strike across for NA]

105) q:4ed
Do you have any biological children?

Mark one:
- ❍ a) Yes
- ❍ b) No

Or: DK / RF / MI [strike across for NA]

106) q:4eg
IF YES:
How many years old is your youngest child?

______

Or: DK / RF / MI [strike across for NA]

107) q:4ht
Would you like to have (a/another) child, or would you prefer not to have any (more) children?

Mark one:
- ❍ a) Have (a/another) child
- ❍ b) No more / none
- ❍ c) Says she can't get pregnant

Or: DK / RF / MI [strike across for NA]

Continue on next page ➔

DCE I
Sociodemographics
Page **12** of **13**
ef029f81b850d61fd9c40c8040fff17f522d9402

HCT-DCE v50, created 10 Sep 2012 ▶ Tracking #: __ __ __ __

q:4fl

Socioeconomics

108) q:4i7

TYPE OF RESIDENCE

Mark one:

❍ a) Room(s)
❍ b) House
❍ c) Apartment
❍ d) Other ____________

Or: DK / RF / MI [strike across for NA]

109) q:4hu

MAIN MATERIAL OF THE FLOOR

Mark one:

❍ a) Earth
❍ b) Cement
❍ c) Other

Or: DK / RF / MI [strike across for NA]

110) q:4dq

Does your household have:

Mark all that apply (read ONE-by-ONE):

❒ a) Electricity
❒ b) A radio?
❒ c) A television?
❒ d) A mobile telephone?
❒ e) An iron?
❒ f) A refrigerator?
❒ g) A motorcycle or motor scooter?
❒ h) A car or truck?
❒ i) A bank account?
❒ j) Tap water?
❒ k) None of the above

Or: DK / RF / MI [strike across for NA]

111) q:4hv

What kind of toilet facility do members of your household usually use?

Mark one (READ responses aloud):

❍ a) Flush toilet
❍ b) Pit latrine
❍ c) Other

Or: DK / RF / MI [strike across for NA]

112) q:4fz

What is the tenure status of the main residence?

Mark one (READ responses aloud):

❍ a) Owner occupied
❍ b) Employer owned
❍ c) Rented
❍ d) Other ____________

Or: DK / RF / MI [strike across for NA]

113) q:4f5

Do you watch television …

Mark one (READ responses aloud):

❍ a) Almost every day
❍ b) At least once a week
❍ c) Less than once a week
❍ d) Not at all

Or: DK / RF / MI [strike across for NA]

114) q:4eb

Are you currently covered by any health insurance?

Mark one:

❍ a) Yes
❍ b) No

Or: DK / RF / MI [strike across for NA]

☞ q:4jg

Thank you very much.

115) q:4jd

TODAY'S DATE:

Year: _____ / Month: _____ / Day: _____

Or: DK / RF / MI [strike across for NA]

116) q:4je

TIME:

____

Or: DK / RF / MI [strike across for NA]

117) q:4jf

INTERVIEWER SIGNATURE:

____

Or: DK / RF / MI [strike across for NA]

End of survey.

DCE I
Socioeconomics
Page **13** of **13**
ef029f81b850d61fd9c40c8040fff17f522d9402
