# Supplementary material for: Heterogeneous HIV Testing Preferences in an Urban Setting in Tanzania: Results from a Discrete Choice Experiment
Source: PLoS One. 2014 Mar 18;9(3):e92100. doi: 10.1371/journal.pone.0092100 (PMC3958474; doi:10.1371/journal.pone.0092100)
Supplement: Appendix S2 — Validity checks. (DOC) [file pone.0092100.s002.doc]

**Heterogeneous HIV testing preferences in an urban setting in Tanzania: Results from a Discrete Choice Experiment**

**Appendix 1: Validity checks**

Literacy and visual acuity. At the beginning of the iPad-based survey, participants were asked to read 3 short sentences in 25 point, 18 point, and 12 point font sizes, the same sizes as those used in the descriptions of DCE attribute levels. Those unable to read the sentence in 25 point font were given an adaptation of the E-test [^55^](#_ENREF_54) to assess their visual acuity. The E-test asks participants to describe the orientation of ‘E’s of different sizes pointing in different directions. All three persons who were unable to read the sentence in 25 point font passed the E-test. Two persons who were not able to read sentences in 18 and 12 point font, respectively, were considered eligible to participate as they were able to read the main attribute descriptors in 25 point font.

***Comprehension test:*** Each participant was given a comprehension test, in which a dominant alternative was created by combining the participant’s most preferred attribute levels, and a dominated alternative was created by combining the participant’s least preferred attribute levels. Asked to choose between these alternatives, eight participants (1.6%) failed the comprehension test by choosing the dominated alternative. These participants were excluded from the analysis.

***Response biases:*** There was no evidence of systematic left-right biases in participants’ choice patterns. Averaging across the 9 choice tasks, participants chose the left alternative 4.49 times, and the right alternative 4.51 times (*t*-test, p=0.931). The distributions of individual participants’ left and right choice frequencies closely resembled a normal distribution with a skewness of 0.06 and a kurtosis of 2.76. Four persons (0.8%) chose always A or always B.

A conditional logit model with interactions between attribute levels and a binary indicator for the alternate attribute order (administered to 128 study participants) assessed whether the order in which attributes were introduced may have influenced their relative importance. For females, none of the 9 interaction parameters (14 with reference terms) were significant. Among males, the alternate order (testing days, type of sample, medication availability, distance, confidentiality) was associated with lower preferences for venipuncture (p=0.010) and a stronger preference for the spouse knowing (p=0.006) as opposed to no-one knowing (p=0.043) about the HIV test. While it cannot be ruled out that this interaction effect is related to the confidentiality attribute being introduced last, the lack of systematic associations among females and the lack of other systematic associations with attribute order among males led us to conclude that attribute order did not meaningfully impact participants’ prioritization of attributes.

***Dominance:*** The distribution of participants’ most and least preferred attribute levels (Table 1) and the preference heterogeneity observed in the mixed logit results (Table 3) suggest that there is no natural ordering of levels within attributes. Thus dominance was assessed not as commonly implemented for each attribute, but separately for each attribute level. Specifically, dominance was assessed by rates of participants who always selected an attribute level when given the option, and/or never chose an attribute level unless forced to by overlap. Between 3 and 22 percent of participants consistently chose one attribute level; rates were highest for distance to testing (16% always chose testing at home, 13% in the neighborhood, and 14% elsewhere in town) and the method for obtaining the sample for HIV testing (14% always chose venipuncture, 22% finger pricks). Across participants, 35% had one, 26% two, and 15% three or more potentially dominant attribute levels. Nearly half (44%) of participants never chose testing out of town, 10% and 22%, respectively, never chose testing at home or in their neighborhood, and 19% never chose oral testing.

***Interactions:*** To test for interactions between attributes, a main effects mixed logit model on all participants was repeatedly estimated with every possible two-way interaction between the levels of different attributes. Main effects were specified as correlated random coefficients, interaction terms as fixed parameters. Wald tests assessed the statistical significance of interaction terms. Only 3 of 77 interactions (including derived interactions for the reference levels) were statistically significant: (1) testing at home x spouse will know (p=0.007); (2) testing in the neighborhood x venipuncture (p=0.026); and (3) testing elsewhere in town x oral swab (p=0.01). The interaction between medication availability and distance to testing, incorporated in the updated experimental design, was not significant.
